# The last 3 decade of vaccination coverage in the Balkan and Eastern Europe countries with reference to the impact of the COVID-19 pandemic

**DOI:** 10.3389/fphar.2024.1278771

**Published:** 2024-06-06

**Authors:** Milos Stepovic, Viktorija Dragojevic Simic, Ivana Zivanovic Macuzic, Radoje Simic, Stefan Vekic, Marija Sekulic, Snezana Radovanovic, Milena Maricic, Marija Sorak, Vesna Suljagic, Radisa Vojinovic, Nemanja Rancic

**Affiliations:** ^1^ Department of Anatomy, Faculty of Medical Science, University of Kragujevac, Kragujevac, Serbia; ^2^ Centre for Clinical Pharmacology, Military Medical Academy, Belgrade, Serbia; ^3^ Medical Faculty of the Military Medical Academy, University of Defence, Belgrade, Serbia; ^4^ Department for Plastic Surgery, Institute for Mother and Child Healthcare of Serbia Dr. Vukan Cupic, Belgrade, Serbia; ^5^ Faculty of Medicine, University of Belgrade, Belgrade, Serbia; ^6^ Faculty of Economics, University of Belgrade, Belgrade, Serbia; ^7^ Department of Hygiene and Ecology, Faculty of Medical Sciences, University of Kragujevac, Kragujevac, Serbia; ^8^ Department of Social Medicine, Faculty of Medical Sciences, University of Kragujevac, Kragujevac, Serbia; ^9^ The College of Health Sciences, Academy of Applied Studies Belgrade, Belgrade, Serbia; ^10^ Department of Gynecology and Obstetrics, Faculty of Medical Sciences, University of Kragujevac, Kragujevac, Serbia; ^11^ Department of Healthcare-Associated Infection Prevention and Control, Military Medical Academy, Belgrade, Serbia; ^12^ Department of Radiology, Faculty of Medical Sciences, University of Kragujevac, Kragujevac, Serbia

**Keywords:** global health, vaccination, prevention, health system, COVID-19

## 1 Introduction

Primary healthcare is the basis of the global health pyramid of every country. The good organisation of primary healthcare influences the national health organisation and it is directly connected to the individuals’ health and theirs healthcare utilisation (van Weel and Kidd, 2018). Reaching the universal health coverage and empowering the primary healthcare are the biggest interests of every global-health policy initiative (World Health Organization, 2017). United Nations’ 2015 Sustainable Development Goals presents a concept of universal health coverage with main goals of giving the people high-quality services that are effective, safe and very important—affordable, both medicines and vaccines, and other services necessary to reach the best of health, ensuring people’s healthy and long lives (Sustainabledevelopment, 2015; M et al., 2022; Jha et al., 2016; Stigler et al., 2016; Gizaw et al., 2022).

Vaccination against measles, mumps and rubella is carried out in children from the 12 until the 15 months of age, using the MMR vaccine. The second dose of the MMR vaccine is given when enrolling in primary school. Two doses of the vaccine are necessary for protection. They provide long-lasting immunity. Immunity after vaccination is created in more than 95% of the vaccinated and is considered lifelong (Centers for Disease Control and Prevention, 2021). Active immunization against polio is carried out in children older than 2 months. The first revaccination is carried out in the second year of life; the second revaccination is carried out before enrolment in the first grade of primary school (Centers for Disease Control and Prevention, 2022).

Today, all countries of the world implement systemic immunization against measles in order to protect both individuals and the population, as a whole. Due to the small number of children who received the vaccine a real threat of a measles epidemic exists, since only when 95 percentages of them have been vaccinated, it can be considered that sufficient collective protection has been provided to prevent an epidemic (Signorelli et al., 2018). Since 2018, compulsory vaccination against mumps-measles-rubella has been introduced in 9 European countries (Bulgaria, Croatia, Czech Republic, France, Hungary, Italy, Latvia, Poland, Slovakia) and against poliomyelitis in 10 countries (Belgium, Bulgaria, Croatia, Czech Republic, France, Hungary, Italy, Latvia, Poland, Slovakia) (Bozzola et al., 2018).

As the part of the primary healthcare, the effective services for immunization must be available to all people which will have positive impact to the universal health coverage. Good health organisation also means the sufficient number of educated and motivated medical workers (World Health Organization, 2030). Pharmaceutical companies are also important part of the whole process, and their supply with high-quality vaccines and sufficient quantity is crucial, as it was during the global COVID-19 pandemic that started in 2019, but proclaimed as the global pandemic in 2020 (Farlow et al., 2023). Situation like this is very good indicator of accessing the effectiveness of health system and primary healthcare. Indicators that are standardized and measures the results of primary healthcare should be used when undergoing the health system reforms (Brisse et al., 2020). During the last 4 years, COVID-19 has changed the system of the healthcare in many ways but also established the imperative for accelerated progress in infodemic management and that is especially important in considering the problem of implementing vaccination (Zielinski, 2021).

Countries that have been chosen for investigations have a historically similar background and as so, very similar ideas for organisation of health systems. There are several health systems, but first originally comes from the Russia (former USSR) and all new ones are modified version. Also, considering this similar background, these countries have a wide diversity in the religion aspects, which is one of reasons how some countries navigates their approaches towards important solutions compared to other countries (Stepovic et al., 2023).

The aim of this paper is to analyse the vaccination coverage in Balkan and Eastern European countries that share similar background in healthcare system organisation; to assess their achievement through the last 3 decades as well as to predict the vaccination coverage until 2025. Aspect of influence of COVID-19 pandemic on the vaccination coverage has also been discussed along with the adaptability of primary healthcare organisation towards the global health emergency. The beginning of the COVID pandemic can reflect the capability of countries to adapt to global alerts and like that, it can describe the stability of the health systems of the countries.

## 2 Methodology

This is an epidemiological, retrospective and descriptive study based on the data of the national population for the selected countries of our research interest. Data is extracted from the European Health for All databases (HFA-DB), where WHO European Region is reporting important statistics related to the health (World Health Organisation, 2023). This database consists from different indicators that are based on reports, and are part of the major monitoring frames (last updated on September the first, 2022). Indicators of interest for this research were following indicators: Percentage of infants vaccinated against polio (% of infants who reached their first birthday in a given calendar year and who were fully vaccinated against polio (3 doses)); Percentage of infants vaccinated against rubella (% of infants who reach their second birthday in a given calendar year and who are fully vaccinated against rubella), and percentage of children vaccinated against measles (% of children up to their second birthday who are fully vaccinated against measles (1 dose)).

The data are anonymous and do not belong to individual citizens, so there is no concern of data privacy protection. The research does not require consideration by the Ethics Committee as it is not a clinical/experimental interventional study involving humans, according to the International Ethical Guidelines for Biomedical Research Involving Humans as well as WHO Good Clinical Practice (World Health Organization, 2005; Council for International Organizations of Medical Sciences, 2016).

The countries of interest that are observed are: Albania, Bosnia and Herzegovina, Bulgaria, Greece, Croatia, Montenegro, North Macedonia, Romania, Serbia, Slovenia, Turkey, Russia, Belarus, Lithuania, Latvia, Estonia and Ukraine. The observation time period is in the range from 1990 to 2020.

The method of univariate analysis was used for a descriptive presentation such as tabulation and graphic presentation of individual characteristics of the observed variables. Indicators of dispersion (range and interquartile difference) and central tendency (median values) were also calculated. The operations were calculated for each country and indicator.

Indicators that we used are tracked only through time so continuous variable was suiting to be performed a statistical method of a linear trend. We calculated the current linear trends and regression by using the IBM SPSS software package Version 26.0. (The Statistical Package for the Social Sciences software) (Version 26.0., SPSS Inc., Chicago, IL).

A regression uses following formula to calculate its predictions: Y = A+ BX.

Dependent variable is Y, independent variable is X, B is the slope of the line and A is the point where Y intercepts the line.

Regression also gives an R-squared value; the values range from 0 to 1. Regression trend can be described as positive or negative. The confidence interval for prediction was 95%.

Forecasting analysis was performed by combining Excel analysis and IBM product designed for statistical and predictive analysis with an aim to access the future changes in the value of the indicators according to the current trend, to the year of 2025 which is considered as the medium term of forecasting; smaller prediction in the contrary to the bigger term prediction are recommended as more accurate.

The data set taken from Health for All, provided the dates up to the year 2019/2020 which was well-suited for comparison of the influence of the beginning of the COVID pandemic on the vaccines that are, in most countries, obligated and recommended being covered at the high percentages (at the level of eradication).

## 3 Results

### 3.1 Percentage of children vaccinated against measles

**GRAPH 1:**
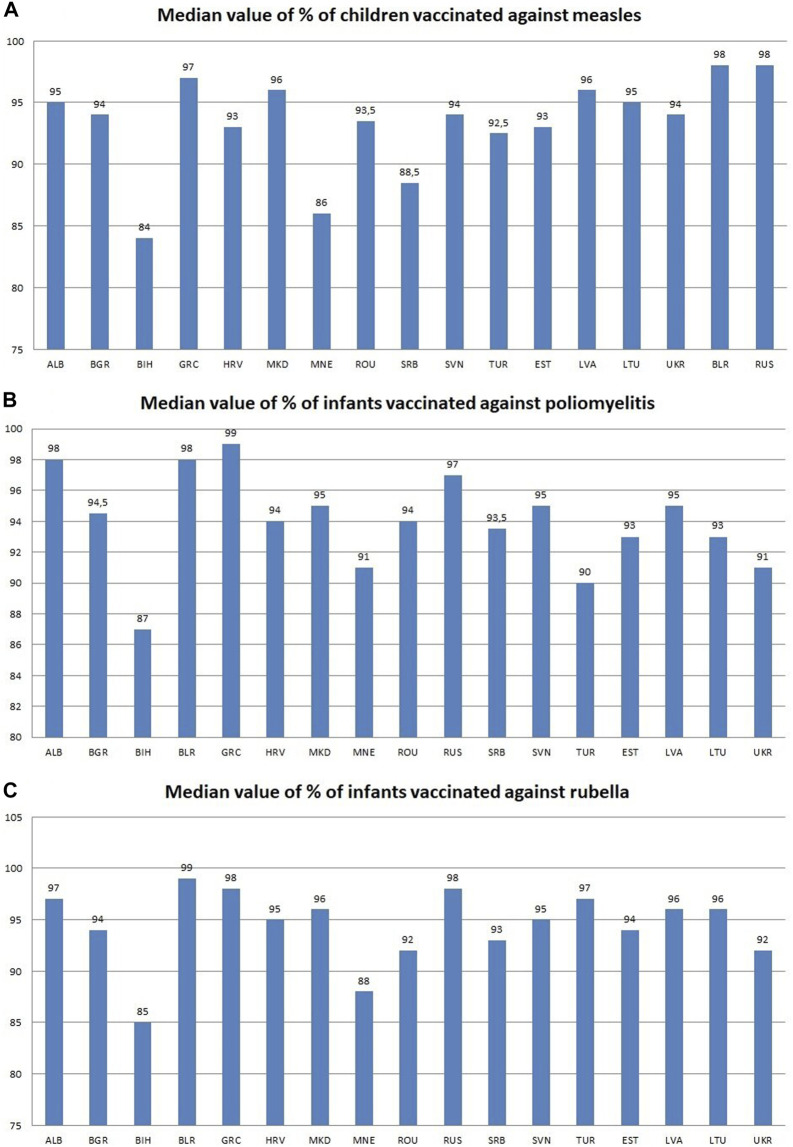
**(A)** Median value for the indicator Percentage of children vaccinated against measles; **(B)** Median value for the indicator Percentage of infants vaccinated against poliomyelitis; **(C)** Median value for the indicator Percentage of infants vaccinated against rubella; Albania—ALB, Bosnia and Herzegovina—BIH, Bulgaria—BGR, Greece—GRC, Croatia—HRV, Montenegro—MNE, Northern Macedonia—MKD, Romania—ROU, Serbia—SRB, Slovenia—SVN, Turkey—TUR, Russia—RUS, Belarus—BLR, Lithuania—LTU, Latvia—LVA, Estonia—EST, Ukraine—UKR.

Russia and Belarus have the highest median indicators of the percentage of children vaccinated against measles with 98% of vaccinated children, while the lowest values are in Bosnia and Herzegovina with 84% and Serbia with 88.5% (Graph 1A). Regression analysis shows an increase in the percentage of vaccinated children in most countries, with the exception of 6 countries, most prominently were Montenegro (y = −4.8143x + 109.05; *R* ^2^ = 0.812), Ukraine (y = −0.9023x + 100.97; *R* ^2^ = 0.2192) and Romania (y = −0.243x + 96.81; *R* ^2^ = 0.2583), in which the percentages are decreasing (Table 1). Compared to the last observed year, the percentage of vaccinated children against measles is expected to increase in the 13 observed countries by the year 2025, with the largest increase in Bosnia and Herzegovina, by 15% more, while the largest decrease in the percentage of vaccinated children is expected in Montenegro and Ukraine by 22% and 17%, respectively.

**TABLE 1:** Values of the indicators: a) Percentage of children vaccinated against measles - F - First year of follow-up (1991), L - Last year of follow-up (2019/2020), P - Prediction to 2025 and LR - linear regression analysis trend; b) Percentage of vaccinated newborns against poliomyelitis - F - First year of follow-up (1990/1991), L - Last year of follow-up (2019/2020), P - Prediction to 2025 and LR - linear regression analysis trend; c) Percentage of infants vaccinated against rubella - F - First year of follow-up (2000), L - Last year of follow-up (2018), P - Prediction to 2025 and LR - linear regression analysis trend.

Countries		F	L	P	LR		F	L	P	LR		F	L	P	LR
Albania	**a) Percentage of children vaccinated against measles**	80	95	100	positive	**b) Percentage of vaccinated new-borns against poliomyelitis**	82	99	100	positive	**c) Percentage of infants vaccinated against rubella**	93	94	96	positive
Bulgaria		98	88	93	negative		99	91	91	negative		89	93	91	positive
Bosnia and Herzegovina		95	68	83	positive		93	73	84	positive		88	68	55	negative
Belarus		95	97	99	positive		90	97	99	positive		98	98	98	negative
Greece		72	97	100	positive		96	99	100	positive		89	97	97	positive
Croatia		89	93	94	positive		85	94	97	positive		94	93	89	negative
North Macedonia		93	75	84	negative		94	92	94	negative		97	75	74	negative
Montenegro		N/A	24	12	negative		90	84	80	negative		90	42	25	negative
Romania		92	87	87	negative		92	87	86	negative		91	90	86	negative
Russia		79	97	100	positive		69	97	100	positive		13	98	100	positive
Serbia		76	87	90	positive		81	97	98	positive		89	93	83	negative
Slovenia		81	94	95	positive		95	95	95	negative		95	93	92	negative
Turkey		73	95	100	positive		74	98	100	positive		98	96	96	negative
Estonia		78	91	96	positive		68	91	99	positive		95	93	91	negative
Latvia		96	99	98	positive		92	99	96	positive		97	98	95	negative
Lithuania		86	93	92	negative		77	91	95	positive		97	92	90	negative
Ukraine		89	85	68	negative		81	84	57	negative		71	91	62	negative

### 3.2 Percentage of infants vaccinated against poliomyelitis

The percentage of vaccinated newborns against poliomyelitis has the highest median value in Greece (99%), Albania and Belarus with 98% of vaccinated children, while the lowest median value have Bosnia and Herzegovina and Turkey with 87% and 90% of vaccinated children, respectively (Graph 1B). The regression analysis shows that the majority of observed countries shows a positive trend concerning the number of vaccinated infants, with the largest increase in Turkey (y = 0.921x + 73.135; *R* ^2^ = 0.7361) and Russia (y = 0.8016x + 79.11; *R* ^2^ = 0.5274) (Table 2). On the contrary, negative trend can be observed in 6 observed countries, with most prominent decrease in Montenegro (y = −0.6143x + 95.248; *R* ^2^ = 0.5763) and Ukraine (y = −1.141x + 102.32; *R* ^2^ = 0.3362) The percentage of infants vaccinated against polio by 2025 will increase in 11 observed countries, with the largest expected increase in Bosnia and Herzegovina, by 11% compared to last observed year. In 4 countries, a decrease in the percentage of vaccinated people is predicted; the highest in Ukraine, by 27%, while in Bulgaria and Slovenia the percentage is predicted to have no change until 2025.

### 3.3 Percentage of newborns vaccinated against rubella

The percentage of newborns vaccinated against rubella with the highest median value, in the observed period, have Belarus with 99% of vaccinated newborns, followed by Russia and Greece with 98% vaccinated in both, while Bosnia and Herzegovina has the lowest median value with 85% of registered users and Montenegro with 88% (Graph 1C). The regression analysis shows that the majority of observed countries show a negative trend in the number of vaccinated newborns, in 13 of the 17 observed countries, while a positive trend can be observed in Russia (y = 2.4691x + 64.286; *R* ^2^ = 0.3805), Albania, Bulgaria and Greece (Table 3). The percentage of infants vaccinated against rubella by 2025 will decrease in 12 countries, most notably in Ukraine (by 29%) and Montenegro (by 17%) when compared with year of 2018. The increase in the percentage of people vaccinated is predicted only in 2 countries, in Russia, where a 100% vaccination rate is expected, as well as in Albania (96%).

## 4 Discussion

Speaking about measles vaccination, its mean coverage decreased from 2009 (94.9%) to 2017 (93.9%) in Europe (European Centre for Disease Prevention and Control, 2018). The highest drop in coverage was in the year 2010–90.4%. However, the vaccination rate in 2017 was below 90% in Romania, Croatia, Cyprus and France (Rota et al., 2016). In our research, Belarus and Russian Federation had the highest median value of vaccination coverage (98%), in Bosnia and Herzegovina it accounted only 84%, while the regression analysis indicated that a largest decline in the measles vaccination rate was in Montenegro. Considering that there is a lot of prejudice in the world about vaccination, especially about vaccine safety and efficacy, this barrier can lead to refusal of vaccination in some countries, making difficult eradication of measles and rubella (O'Connor et al., 2017; Cutts et al., 2021). In our research, a trend of declining rubella vaccination was found in 13 countries, with largest decline in the Estonia. Only 4 countries showed positive trend in the regression analysis concerning rubella vaccination, what is highly concerning fact.

Most countries in the European region have achieved financial self-sufficiency for vaccines, and the remaining challenge in most countries is the allocation of additional financial resources to expand immunization programs (World Health Organization, 2014). According to an expert review of vaccination, Bechini et al., 2019 found that countries in the European region, such as Greece, Hungary and Luxembourg, have a positive trend in polio vaccination and reported the highest coverage rates (99%) in 2017. On the other hand, Poland and Romania reported a negative trend and recorded the lowest coverage (82%) in 2017 (Funk et al., 2019). In our study, it was also found that among selected countries, Greece has the highest polio vaccination coverage (99%), and positive trend could be observed in the largest number of selected countries (11 out of 17).

Comparing the first and the last available years of vaccination percentages it can be clearly seen that Bulgaria, Bosnia and Herzegovina, North Macedonia, Montenegro and Romania had a prominent decrease in the number of vaccinated children for measles and poliomyelitis. As the last available year for the vaccination was 2020—the year when the pandemic was globally proclaimed, we can assume that this was partly due to the weaker organisation of health system of these countries that affected the primary healthcare on the different levels. Reluctance toward health services has been reported in past epidemics, and apparently the COVID-19 pandemic is not an exception (van Stekelenburg et al., 2022). Along with the epidemic of COVID-19, the infodemic spread. Fear of contagion mixed with lack of information concerning both vaccination and COVID‐19 raised the vaccine hesitancy (Hudson and Montelpare, 2021). Severe shortages of healthcare providers and disruptions in the supply chain due to border closures and travel restrictions, contributed to disrupted immunization programs in many countries, especially developing ones (Moosavi et al., 2022). However, these results are not accidental. For example, in Romania, measles remains endemic, and difficulties in delivering vaccine doses, sometimes lack of sufficient vaccine supplies and absence of a legislation concerning immunization are some of the main obstacles (Dascalu, 2019). In Bosnia and Herzegovina, the situation related to vaccination has also been unsatisfactory for some time (UNICEF for every child, 2023). Not only that Bosnia and Herzegovina had a severe outbreak of measles in 2014 in Central Bosnia Canton with 5048 cases, but also it is one of 4 countries in Europe, together with Romania, Ukraine, and Georgia under high risk of poliovirus intrusion. The situation in Serbia is also complex. In the two measles epidemics registered in the period 2014-2015 (Medić et al., 2019), as well as in the period 2017-2019 (UNICEF for every child, 2022b), thousands of infected and hospitalized and dozens of deaths were registered.

In the WHO European region in 2022, DTP vaccine had average 94% coverage of diphtheria, tetanus and pertussis in comparison to the 93% of coverage with first doses of measles vaccine. This is important because of the decline in vaccination that occurred during the COVID-19 pandemic, and almost half of countries in this region did not returned to the level of coverage of those vaccines, that they had before pandemic happened—in the early 2019. Our work is in the line with statement about declining for the coverage of measles, as it was noticed in the 5 countries and prediction to 2025 showed that 6 counties will continue to have decreeing trend.

Although the situation in 2021 in comparison to 2020 was promising, it is still not good enough. The situation is especially complicated in Montenegro and Ukraine. According to the Montenegro government data immunization coverage rates for the first dose of measles, mumps and rubella have decreased from 90% in 2010 to a 23.88% among children born in 2019, who were supposed to receive their first dose of the measles vaccine during 2020 (UNICEF for every child, 2022a). Our results concerning regression analysis also showed decrease in the percentage of vaccinated children in 6 countries, but most prominently in Montenegro, Ukraine and Romania. Unfortunately, large health crises like COVID-19 pandemic are not the only ones who can seriously endanger not only immunization process, but also national health organisation as a whole. Namely, the last outbreak of measles in Ukraine occurred in 2017–2019, but now there is a risk of another serious outbreak, since the war with Russia worsened the situation as a result of destroyed large infrastructure systems, including medical one (Burynska, 2023).

The reflection of the approach to the vaccination in general, between the higher and lower income countries can be described with a fact about the vaccination for COVID-19 from Our world in data, where 80% of higher income counties received the first does and only 23% of the lower income counties (Maltezou et al., 2022). Overall, a significant drop in the vaccination rate, especially in the low-income countries, may result in dangerous sequels regarding children health. It can potentially increase the mortality and morbidity from polio, measles, and other vaccine-preventable diseases, particularly in countries with low coverage (Macdonald et al., 2020; Seyed et al., 2022). Some European countries and most of Balkan countries have a huge problem with vaccination coverage because of the misinformation about the safety of the vaccine and their efficacy, and that’s a problem that health organisation must know because the fear is the root of most problems (Cvjetkovic et al., 2022; Fan et al., 2022). Immunization programs should be strengthened within primary healthcare (Effraimidou et al., 2023) Countries should also implement robust surveillance systems to identify and close immunity gaps (Blanc et al., 2022).

### 4.1 Limitation and strengthen of study

We have presented the vaccination coverage against measles, rubella and polio viruses—the vaccines that are given in the early children’s age in first few months or years, as those vaccinations coverage were the most discussed in topics considering the influence of COVID-19 pandemic. The assessment of vaccination coverage in Balkan and Eastern European counties that have shared historic background and similar healthcare system organization haven’t been analysed in this perspective along with describing the effect of pandemic. Limitation of our study was not involving the other types of vaccine, like DTP (diphtheria, tetanus, and pertussis) vaccine that is also given in the childrens age. The analysis of these vaccines is encouraged to be done in the future because it is from the public health importance.

## 5 Conclusion

Indicators of vaccination percentage for measles, rubella and poliomyelitis were compared for the seventeen countries in the Balkan and Eastern Europe. Compared to the last observed year, the percentage of vaccinated children against measles is expected to increase in the 13 observed countries by the year 2025. The majority of observed countries also show a positive trend concerning the number of vaccinated infants against poliomyelitis, while in 4 countries, a decrease in the percentage of vaccinated people is predicted; the highest in Ukraine, by 27%. On the contrary, the increase in the percentage of people vaccinated against rubella is predicted only in 2 countries, in Russia, as well as in Albania. However, analysis of the influence of COVID-19 pandemic on the vaccination coverage reveals that it was most prominent in countries which already had problems with national program of immunization.Studies like this are necessary for the better organisation of health systems and primary healthcare which can benefit the universal health coverage.

## Data Availability

Publicly available datasets were analyzed in this study. This data can be found here: https://gateway.euro.who.int/en/datasets/european-health-for-all-database.
